# Molecular Dynamics Simulation on Creep Behavior of Nanocrystalline TiAl Alloy

**DOI:** 10.3390/nano10091693

**Published:** 2020-08-28

**Authors:** Fei Zhao, Jie Zhang, Chenwei He, Yong Zhang, Xiaolei Gao, Lu Xie

**Affiliations:** 1 National Center for Materials Service Safety, University of Science and Technology Beijing, Beijing 100083, China; zhaofei@ustb.edu.cn; 2 Key Laboratory of Fluid Interaction with Material, Ministry of Education, Beijing 100083, China; 3 School of Mechanical Engineering, University of Science and Technology Beijing, Beijing 100083, China; zhangjie_043@163.com (J.Z.); 18832049011@163.com (X.G.); 4 China Nuclear Power Technology Research Institute Co., Ltd., Reactor Engineering and Safety Research Center, Shenzhen 518031, China; 5 Beijing Advanced Innovation Center of Materials Genome Engineering, State Key Laboratory for Advanced Metals and Materials, University of Science and Technology Beijing, Beijing 100083, China; drzhangy@ustb.edu.cn

**Keywords:** molecular dynamics simulation, creep behavior, nanocrystalline, TiAl alloy

## Abstract

TiAl alloy represents a new class of light and heat-resistant materials. In this study, the effect of temperature, pressure, and grain size on the high-temperature creep properties of nanocrystalline TiAl alloy have been studied through the molecular dynamics method. Based on this, the deformation mechanism of the different creep stages, including crystal structure, dislocation, and diffusion, has been explored. It is observed that the high-temperature creep performance of nanocrystalline TiAl alloy is significantly affected by temperature and stress. The higher is the temperature and stress, the greater the TiAl alloy’s steady-state creep rate and the faster the rapid creep stage. Smaller grain size accelerates the creep process due to the large volume fraction of the grain boundary. In the steady-state deformation stage, two kinds of creep mechanisms are manly noted, i.e., dislocation motion and grain boundary diffusion. At the same temperature, the creep mechanism is dominated by the dislocation motion in a high-stress field, and the creep mechanism is dominated by the diffusion creep in the low-stress field. However, it is observed to be mainly controlled by the grain boundary diffusion and lattice diffusion in the rapid creep stage.

## 1. Introduction

TiAl alloy exhibits the advantages of low density, high stiffness, heat-resistance, and excellent anti-oxidative character. In addition, it has superior mechanical properties at high temperatures, enabling its use at temperatures ranging from 700 °C to 1000 °C, making it one of the excellent candidates for high-temperature structural materials in contemporary aerospace, ordnance, and civil industries [1,2,3]. Thus, TiAl has vital engineering application potential. Up to now, it has been successfully applied in the engine blades of the aerospace vehicles [4], exhaust valves of the vehicle engines [5,6], and rotors of the turbocharger [7,8]. At the same time, significant research achievements have also been attained with respect to the mechanical properties and deformation mechanisms at high temperatures. Kad B K et al. [9] evaluated the ductile deformation behavior of TiAl alloy at high temperature and concluded that the dislocation climb contributed significantly to the plastic deformation and promoted the deformation of TiAl alloy at high temperature for the strain rate of 10^−2^ s^−1^ at 900 °C under tensile deformation conditions. Fang Wenbin et al. [10] deciphered the microstructure of TiAl alloy as a (γ/α2 + γ) bi-phase structure using electron microscopy and conducted compression tests at high temperature in an external environment. It was concluded that the dynamic recrystallization is the main softening mechanism of TiAl alloy at high temperatures and a low strain rate. Moreover, the creep behavior is also an important mechanical performance parameter for high-temperature structural materials. Appel F et al. [11] studied the creep strength of TiAl alloy and concluded that the dislocation climbing mechanism controls the creep deformation in the expected temperature range. Also, complex phase transitions were observed during the creep process. Monchoux J P et al. [12] studied the steady-state creep stage of the cast biphasic Ti48-Al48-Cr2-Nb2 (at%) alloy at 750 °C and 150 MPa, and they found that the creep process was controlled by dislocation slip and dislocation climb. Yu Long et al. [13] studied the creep fatigue life of casting Ti-45Al-8Nb-0.2W-0.2B-0.1Y (at%) alloy. They found that the failure life decreased exponentially with the increase of the steady state-creep rate and proposed the linear formula and power law formula of the creep fatigue life.

Molecular dynamics represents a computer-based testing method which can effectively simulate the dynamics behaviors of nanomaterials by taking the microscopic particles as the research object. Up to now, it has been successfully employed to study lattice distortion, grain growth, the stress-strain relationship, diffusion, deposition, nano-friction, atom manipulation, micro-fluids, micro-heat transfer, etc. The molecular dynamics simulation method has also been used to study the deformation mechanism during the different stages of creep deformation of materials. Wang Yunjiang et al. [14] studied the stress-induced transformation of the grain size index during the creep process of nanocrystalline copper via molecular dynamics simulation. It was observed that the grain size index increased with stress and decreased after reaching a critical stress value. The authors proposed a constitutive equation for the creep behavior of nanocrystalline copper controlled by the dislocation nucleation mechanism. Jiao S et al. [15] studied the high temperature creep behavior of polycrystalline nanometer twinning face-centered-cubic metal through molecular dynamics. Strong creep resistance of the nano-twin metal was observed due to the decreased twin spacing under the action of a wide range of the applied stress. In addition, the creep mechanism of the nanocrystalline metal with the high-density twin boundary included grain boundary diffusion and sliding to the point of dislocation nucleation. Md. Meraj et al. [16] studied the creep behavior of nanocrystalline Ni containing the bimodal grain structure. It was discovered that the dislocation density rapidly declined as the creep deformation process progressed, with the grain boundary diffusion taking the lead position in the first and second creep stages. On the other hand, the bimodal nanocrystalline Ni transforms into an amorphous structure during the third stage, and the creep mechanism can be attributed to shear diffusion. S. Pal et al. [17] studied the creep behavior of nanocrystalline Ni and nanocrystalline NiZr with grains of 6 nm size using a molecular dynamics simulation. It was observed through the centrosymmetry parameter that radial distribution function and vacancy defects of the Zr content had a significant effect on the creep properties of nanocrystalline NiZr. Upon increasing the Zr content, the dislocation was observed to be present for a longer period during the creep process. Byungkwan Jeong et al. [18] employed a molecular dynamics simulation to gain insights into the deformation mechanism of TiAl alloy. The results were further analyzed through theoretical predictions, density functional theory (DFT) calculations, and existing tests to verify the rationality of the molecular dynamics simulations for studying the deformation mechanism of the intermetallic compounds. NieKai et al. [19] studied the effects of factors like temperature, stress, and grain size on the high-temperature creep behavior of nanocrystalline Ni by employing molecular dynamics studies. The authors used grain size and the stress index to describe the steady-state creep deformation mechanism and concluded that, upon increasing the temperature and stress level and decreasing the grain size, the creep mechanism was dominated by lattice diffusion and grain boundary diffusion, followed by dislocation nucleation in the last stage.

In this study, the high temperature creep behavior of nanocrystalline TiAl alloy has been studied using a molecular dynamics simulation. The effects of factors like temperature, stress, and grain size on the high temperature creep properties of nanocrystalline TiAl alloy have been studied. The study analyzes the micro- and nano-scale deformation mechanisms during different creep stages, such as crystal structure evolution, dislocation motion, and atomic diffusion. Molecular dynamics suffer from the limitation that the time scale is relatively short, and the system is not large enough. This is different from the actual experiments in the geometric and time scales; thus, the stress level and strain rate obtained by simulation are observed to be much higher than in the actual experiments. Although the creep is a time-related phenomenon beyond the timescale of molecular dynamics, molecular dynamics simulation can enable the observation of the creep phenomenon and microstructure evolution under high strain rates. In addition, the characteristics of the obtained creep curve are the same as the three stages of actual creep: initial creep, stable creep, and accelerated creep. Moreover, the deformation mechanism is also noted to be consistent with the actual high-temperature creep mechanism. Thus, the use of molecular dynamics to study the creep deformation behavior proves the rationality of this method. The time scale and stress level used in this study are similar to the actual tests.

## 2. Model and Method

**Figure 1:**
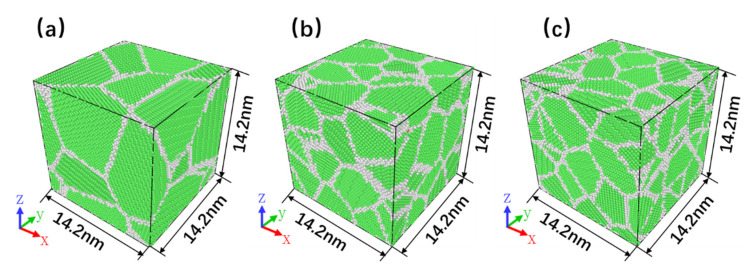
Nanocrystalline TiAl models with different grain sizes of (**a**) 6.6, (**b**) 4.6, and (**c**) 3.8 nm.

The Voronoi algorithm [20] has been used to generate three nanocrystalline TiAl alloy models with different grain quantities in Atomsk [21]. The geometric model of the molecular dynamics simulation is shown in Figure 1, in which the green and grey parts represent the face-centered cubic (FCC) crystal structure and grain boundary, respectively. There are 10, 30, and 50 grains in each model with the grain sizes in the model being 6.6 nm, 4.6 nm, and 3.8 nm, respectively. Moreover, there are 175,132, 175, 183, and 175,152 atoms in the models, with the size of the models being 14.2 nm × 14.2 nm × 14.2 nm.

In this study LAMMPS (Version 2.9.0, Sandia National Labs, Albuquerque, NM, USA) [22], molecular dynamics simulation software, has been used to realize the molecular dynamics simulation calculations of the high-temperature creep process of nanocrystalline TiAl alloy. The visual tool OVITO(Version 3.2.0 of OVITO Basic, OVITO GmbH, Darmstadt, Germany) [23] has been used for structural analysis and processing. In the simulation, the periodic boundary conditions have been used in x, y, and z directions. The potential function for the simulation is the embedding atomic potential (EAM) of TiAl, and this potential describes fundamental material properties of Ti–Al alloys in good agreement with experimental or first-principles data [24]. The time-step was set to be 1 fs. The TiAl nanocrystalline model simulates the creep process for an isothermal and isostatic pressure (NPT) system, in which no pressure is applied in the X and Y directions, whereas a constant creep stress is applied in the Z direction, as per the Parrinello-Rahman method [25]. The creep simulations have been carried out at high temperatures with the simulation lasting 200 ps.

After the simulation, the simulated powder X-ray diffraction patterns (XRDs) were used to analyze the phase of TiAl alloy film, which were determined by using Debye functional analysis as described in detail by Kazakov and co-workers [26,27]. The study has adopted the center symmetry parameters (CSP) [28] to calculate the local atomic damage, thus providing insights into the crystal structure evolution for the high-temperature creep process of the nanocrystalline TiAl alloy. The degree of non-crystallization has been evaluated using the radial distribution function (RDF) during the high-temperature creep process. The dislocation extraction algorithm (DXA) [29] has been employed to analyze the dislocation evolution. The mean square displacement (MSD) [30] has been calculated to judge the atomic diffusion coefficient. Finally, the Wigner—Seitz defect [31] has been used to analyze and calculate the number of vacancies during the creep deformation process.

## 3. Results and Discussion

### 3.1. The Phenomenon and Affecting Factor of Creep Deformation

**Figure 2:**
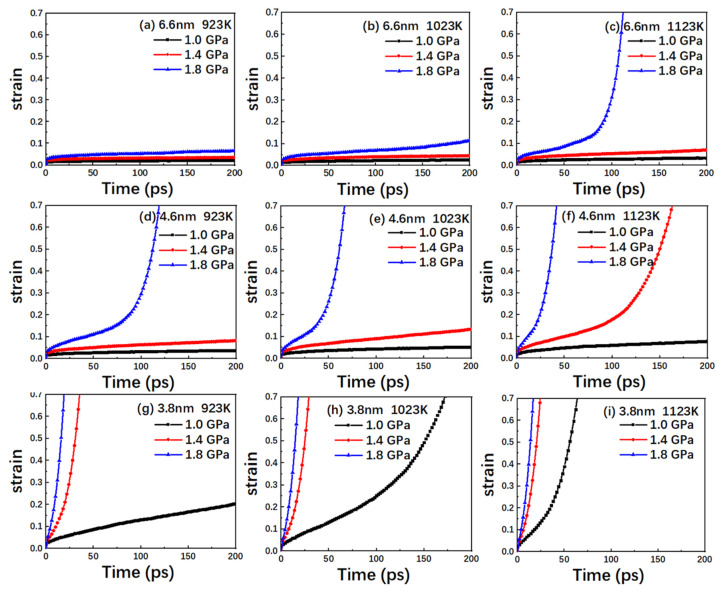
Time versus creep strain curves for the nanocrystalline TiAl models with grain sizes of (**a**) 6.6 nm at 923 K; (**b**) 6.6 nm at 1023 K; (**c**) 6.6 nm at 1123 K; (**d**) 4.6 nm at 923 K; (**e**) 4.6 nm at 1023 K; (**f**) 4.6 nm at 1123 K; (**g**) 3.8 nm at 923 K; (**h**) 3.8 nm at 1023 K; and (**i**) 3.8 nm at 1123 K under the stress levels of 1.0, 1.4 and 1.8 GPa.

Different stresses (1.0 GPa, 1.4 GPa and 1.8 GPa) have been applied at different temperatures (923 K, 1023 K and 1123 K) in the TiAl alloy model with different grain sizes (6.6 nm, 4.6 nm and 3.8 nm) to obtain the variation of strain with time, as shown in Figure 2. In the creep deformation curve shown in Figure 2, the slope represents the strain rate during the creep deformation process. The creep characteristics can be generally divided into three stages: the initial creep stage, where the strain rate gradually decreases on enhancing the duration and finally flattens out; the steady creep stage, in which the strain rate remains constant; and the accelerated creep stage, where the strain rate increases continuously with time.

It can be seen from Figure 2 that the creep curves show initial creep and steady creep stages, while a few of them also exhibit the accelerated creep stage. The model with a grain size of 6.6 nm has a slow creep process, and only a rapid creep stage occurs when the temperature is 1123 K and the stress level is 1.8 GPA. The creep process was relatively accelerated with the grain size of 4.6 nm. At 923 K, only the initial creep and steady creep stages are observed to exist at 1.0 GPa and 1.4 GPa, while the accelerated creep stage appears for 1.8 GPa. In comparison, the creep curve at 1023 K for different stress values is largely the same. However, the strain rate is observed to be higher. At 1123 K, the rapid creep stage appears at both 1.4 GPa and 1.8 GPa, whereas the steady creep rate at 1.8 GPa is noted to be higher than the steady creep rate at 1.4 GPa, with TiAl alloy entering the rapid creep stage earlier. The creep process accelerated with the decrease of grain size. Except for the creep curves of the model with a grain size of 3.8 nm at 923 K and 1.0 GPA, all the other creep curves show a rapid creep stage.

Based on the creep curve, it can be intuitively suggested that increasing the temperature and stress enhances the steady state-creep rate in the high-temperature creep process, thus accelerating the advent of the rapid creep stage and shortening the creep life of TiAl alloy. Besides, decreasing grain size could also accelerate the creep process. The observed phenomena can be explained based on the following three aspects: firstly, as the temperature increases, the vibration frequency and amplitude of the atoms in TiAl alloy exhibit an increase, thus allowing the atoms to pass through the barrier around them. This subsequently accelerates the alloy’s internal diffusion and dislocation. Afterwards, the equilibrium vacancy concentration changes with stress. For the grain boundary and dislocation subjected to the tensile stress, the equilibrium vacancy concentration is noted to increase. Therefore, the vacancy concentration gradient appears in the crystal, with the vacancy diffusing along the fast diffusion path (such as grain boundary and dislocation). At the same time, the diffusion rate may enhance as the atoms are more likely to overcome the barrier at high stress levels. Finally, the grain boundary plays an important role during the creep process of nanocrystalline TiAl, and the decreases in the grain size of a model increase the volume fraction of the grain boundary. Compared with grain interiors, the grain boundary is easier to deform and slide [32]. It is not only a good source or sink place for dislocation and vacancy, but also serves as a fast path for diffusion [33]. Thus, when the GB volume fraction becomes larger in the smaller GS samples, the creep rate increases.

### 3.2. Evolution and Analysis of Crystal Structure

As can be seen from the creep curve in Figure 2, with 4.6 nm grain size and at the stress level of 1.4 GPa, the creep curves associated with 923 K and 1023 K exhibit two steady-state creep stages with different rates, whereas at 1123 K, the associated creep curve exhibits typical creep characteristics. Therefore, the simulation results with 4.6 nm grain size for the different temperatures at 1.4 GPa have been selected to study the evolution of the crystal defects in TiAl alloy.

**Figure 3:**
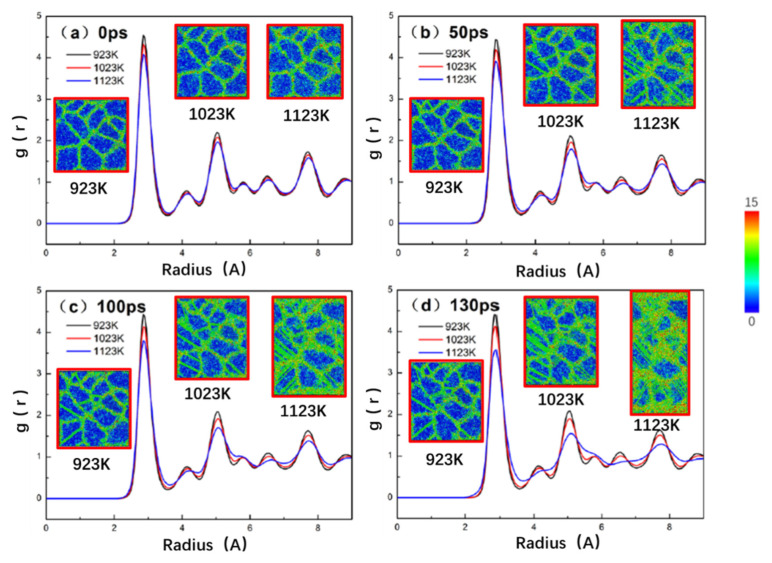
Radial distribution function plots with atomic snapshots of a representative nanocrystalline TiAl model with 4.6 nm coarser grain, colored as per CSP during creep deformation for (**a**) 0, (**b**) 50, (**c**)100, (**d**) 130 ps under the stress levels of 1.4 GPa at 923 K, 1023 K, and 1123 K.

**Figure 4:**
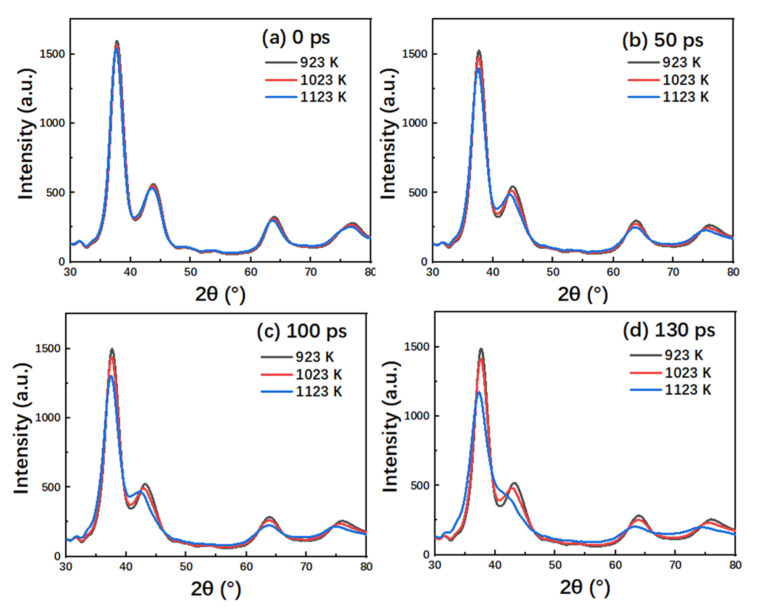
The simulated XRD pattern of a representative nanocrystalline TiAl model with 4.6 nm grain size during creep deformation for (**a**) 0, (**b**) 50, (**c**) 100, (**d**) 130 ps under the stress levels of 1.4 GPa at 923 K, 1023 K, and 1123 K.

Figure 3 shows the radial distribution function (RDF) and corresponding CSP atomic snapshots for the TiAl alloy model with the 4.6 nm grain size at different durations during the creep deformation under 1.4 GPa stress at 923 K, 1023 K, and 1123 K, respectively. In the case of the use of the center symmetry parameters (CSP) to analyze the evolution of the crystal defects, 0 to 15 levels in Figure 4 are usually used for representing the extent of the damage to the regional structure. Corresponding to the atomic snapshot presented in Figure 3, dark blue represents the crystal structure, whereas the green part expresses the grain boundary. The area colored from blue to green to red represents the extent of the damage to the crystal. Combining the creep curve in Figure 2 with the atomic snapshot in Figure 3, it can be observed that the primary and steady-state creep stages with low creep rate occur during the creep process of TiAl alloy at 923 K, and the grains inside the alloy remain largely intact. The creep curve at 1023 K does not show a rapid creep stage; however, the creep rate is noted to increase under the influence of temperature. With the progress of creep deformation, the grain boundary in TiAl alloy becomes wider, with the non-crystallization at the grain edge becoming more obvious than at 923 K. At 1123 K, the creep curve of TiAl alloy presents the three complete creep stages at 50 ps, with the grain non-crystallization being achieved from the grain boundary to the grain interior at 100 ps. Further, TiAl alloy starts to enter the rapid creep stage at 130 ps. At this stage, the grain damage is observed to be serious, with the appearance of even non-crystallization phenomenon indicating that the grains completely disappear and turn into the grain boundary. In addition, the RDF curve is used to determine the atomic sorting state. The peaks in the RDF graph refer to the probability of distribution of the other particles of the same type in space, with a pre-condition that a particle coordinate is known. The spikes represent the ordered arrangement, whereas the round peaks signify disorder. Generally, the first three peaks can be taken into account to determine the atomic ordering state. In the RDF curve shown in Figure 3, the height and shape of the peaks at 923 K and 1023 K do not change much with time and retain the original peak characteristics, thus indicating that the crystal structure of TiAl alloy remains largely stable. In addition, it further signifies that the amorphous phase exists only at the junction of the grains and grain boundary and does not extend into the grain interior during the steady-state creep stage. At 1123 K, the second and third peaks of RDF become significantly smaller and wider after the rapid creep of TiAl alloy. Meanwhile, the fourth and fifth peaks gradually disappear, which indicates that the non-crystallization inside the grains appears and accelerates after the rapid creep stage.

Figure 4 shows the simulated XRD patterns at different times in the creep process of the TiAl alloy at different temperatures. The experimental XRD peaks of TiAl were 37.8°, 43.7°, and 63.7°, respectively [34]. This is corresponding to the peak position in the simulated XRD pattern, while the overall deviation is approximately 1.8°. The reason for the deviation is that MD simulation is limited by the simulation size. In addition, it is not possible to directly compare the strength and width of the XRD peaks of the experimental and simulated samples. The relative strength of the XRD peaks is also related to the crystal quality. At 0 ps and 50 ps, the XRD patterns of the TiAl alloy were almost the same at different temperatures, and the diffraction peak decreased with the increase of temperature, indicating that the FCC phase structure decreased with the increase of temperature. At 100 ps, it was observed that the TiAl alloy was at the end of the steady-state creep stage at 1123 K, and its XRD pattern diffraction peak was slightly lower than that at 923 K and 1023 K, indicating that the FCC phase structure declined slowly in the steady-state creep stage. At 130 ps, the TiAl alloy entered the rapid creep stage at 1123 K, and the maximum diffraction peak was significantly lower than the other two temperature conditions, and the width of the peak became wider, indicating that the crystal structure composition of the TiAl alloy decreased rapidly during the rapid creep stage.

### 3.3. Evolution and Analysis of Dislocation Density

**Figure 5:**
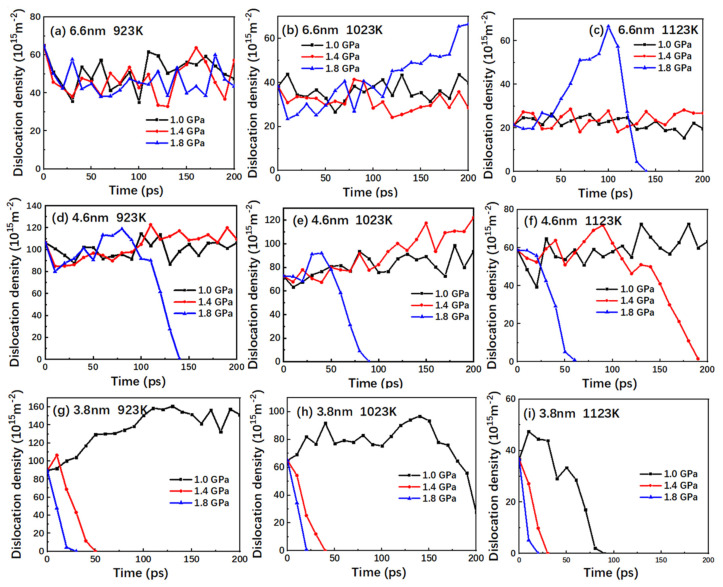
Plots of the dislocation density versus time for the nanocrystalline TiAl models with grain sizes of (**a**) 6.6 nm at 923 K; (**b**) 6.6 nm at 1023 K; (**c**) 6.6 nm at 1123 K; (**d**) 4.6 nm at 923 K; (**e**) 4.6 nm at 1023 K; (**f**) 4.6 nm at 1123 K; (**g**) 3.8 nm at 923 K; (**h**) 3.8 nm at 1023 K; and (**i**) 3.8 nm at 1123 K under the stress levels of 1.0, 1.4, and 1.8 GPa.

During the high-temperature creep, due to the grain boundary defects and stacking faults, dislocation nucleation and annihilation commonly occur. Therefore, the dislocation statistics have been gathered for TiAl alloy at different temperatures and pressures. The corresponding variation of the dislocation density is shown in Figure 5. In Figure 5a, the dislocation density fluctuates steadily at each stress level. In Figure 5b,c, the dislocation density at the stress level of 1.8 GPa shows a gradual increase and a first increase and then a rapid decline, respectively, while the 1.0 GPa and 1.4 GPa are still relatively stable. In Figure 5d, the dislocation density of the alloy at 1.0 GPa and 1.4 GPa is noted to be relatively stable, whereas the dislocation density at 1.4 GPa exhibits a slight increase. On the other hand, the dislocation density at 1.8 GPa is observed to first increase, followed by a rapid decrease. In Figure 5e, the dislocation density at 1.0 GPa is noted to be still relatively flat, while the density at 1. 4 GPa demonstrates an obvious rise. The density for 1.8 GPa presents a brief rise followed by a rapid decline. In Figure 5f, the dislocation density remains stable only at 1.0 GPa, whereas it decreases to zero for both 1.4 GPa and 1.8 GPa, with a much faster decline for 1.8 GPa. In Figure 5g, the dislocation density increases gradually at the stress level of 1.0 GPa and drops rapidly to 0 at the stress levels of 1.0 and 1.4 GPa. In Figure 5h, the dislocation density at the stress level of 1.0 GPa increases slowly at first and then decreases rapidly. In Figure 5i, the dislocation density decreases rapidly to zero at all stress levels.

Combined with the creep curves of Figure 2, the variation in the dislocation density can be explained as follows: during the creep simulation, for the creep curve exhibiting the primary and steady-state creep stages, the dislocation density exhibits a slowly rising trend with the creep deformation on the whole. In addition, the faster the steady-state creep rate changes, the more obvious the increase in the attained dislocation density. For the creep curve in the fast creep stage, the corresponding dislocation density decreases to zero. Moreover, the smaller the change in the steady-state creep stage, the faster the reduction in the dislocation density. This is because, in the fast creep stage, the diffusion of the atoms in the grain boundary causes the model to lose crystallization, thus hindering dislocation motion and eventually eliminating it. Therefore, at this stage, the dislocation mechanism does not drive the creep process. It can be seen that dislocation nucleation and dislocation annihilation play a vital role in the primary and steady creep stages of the creep process, while dislocation is not the dominant factor during the rapid creep stage.

As can be seen with the change of two dislocation density curves at the 1.8 GPa stress level in Figure 5b and in Figure 5c, the growth of the dislocation density at the 6.6 nm grain size was significantly accelerated compared to other grain sizes. This may be because changes in grain size alter the extent of the dislocation slip in grains. Under the action of applied stress, the dislocation source located at the center of the grain starts, the dislocation slips to the grain boundary, and a dislocation plug group is formed in front of the grain boundary [35]. The displacement decreases with the increase of grain size. In addition, when the grain size is large, the inhibition effect of the grain boundary on the proliferation of the dislocation source is weak, and the dislocation source can release a complete stable dislocation loop. When the grain size is small, the hindrance of the grain boundary to the dislocation source increases significantly, and the dislocation source cannot release a stable dislocation loop, which makes the dislocation source unable to operate [35,36].

### 3.4. Creep Mechanism: Diffusion and Vacancy

**Figure 6:**
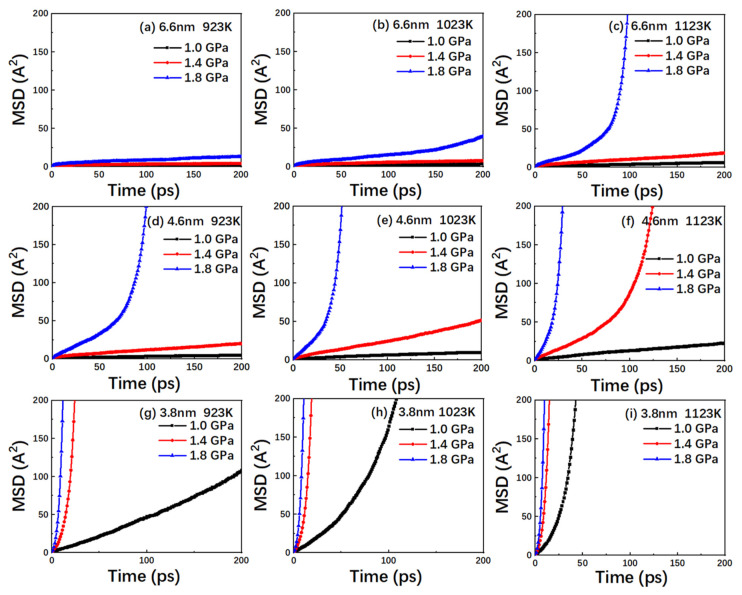
Plots of the MSD versus time for the nanocrystalline TiAl models with grain sizes of (**a**) 6.6 nm at 923 K; (**b**) 6.6 nm at 1023 K; (**c**) 6.6 nm at 1123 K; (**d**) 4.6 nm at 923 K; (**e**) 4.6 nm at 1023 K; (**f**) 4.6 nm at 1123 K; (**g**) 3.8 nm at 923 K; (**h**) 3.8 nm at 1023 K; and (**i**) 3.8 nm at 1123 K under the stress levels of 1.0, 1.4, and 1.8 GPa.

Based on the dislocation density analysis, it is observed that the dislocation disappears in TiAl alloy after entering the rapid creep stage. Therefore, in this study, the MSD curve has been used to determine if the creep process is dominated by diffusion and vacancy. It can be observed from Figure 6 that MSD of TiAl alloy changes as a function of time.

Based on Figure 2, the MSD curve is observed to be similar to the creep curve, thus indicating that the atomic diffusion plays a significantly dominant role in the creep deformation of TiAl alloy. This is mainly due to the fact that the diffusion rate of atoms increases with decreasing grain size and increasing temperature and stress levels. During the evolution of the crystal structure, the extent of amorphization of the grains in TiAl alloy continuously increases during the creep process, which leads to a gradual increase in the proportion of the amorphous components in the model with time. This causes more vacancies in TiAl alloy and, thus, increases the diffusion of atoms. At the same time, compared with the grain interior, the grain boundaries also have high distortional strain energy in addition to a large extent of defects. The diffusion requires activation energy, and distortional strain energy can be used to enrich the activation energy. Thus, the atoms of the grain boundary diffuse swiftly. On the other hand, a decrease in grain size indicates an increase in the volume fraction of grain boundaries in the model, and the atoms located at the grain boundaries exhibit rapid diffusion [32]. In general, the diffusion creep is one of the dominant mechanisms during the high-temperature creep of TiAl alloy.

**Figure 7:**
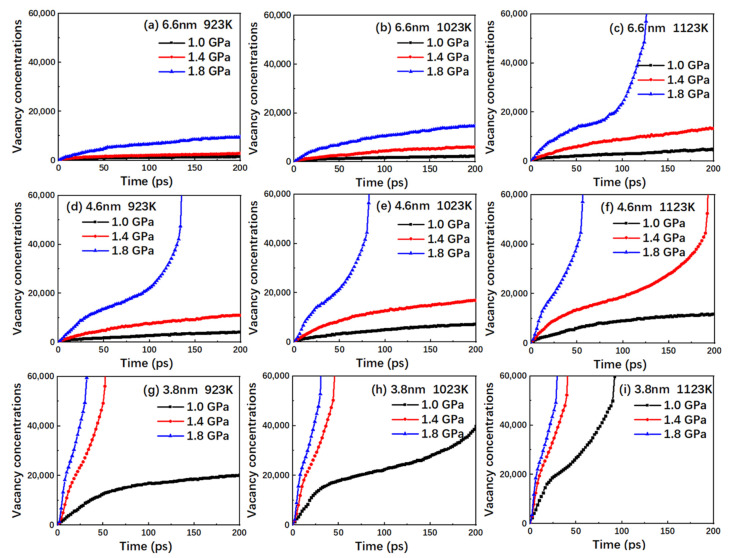
Plots of the vacancy concentrations versus time for the nanocrystalline TiAl models with grain sizes of (**a**) 6.6 nm at 923 K; (**b**) 6.6 nm at 1023 K; (**c**) 6.6 nm at 1123 K; (**d**) 4.6 nm at 923 K; (**e**) 4.6 nm at 1023 K; (**f**) 4.6 nm at 1123 K; (**g**) 3.8 nm at 923 K; (**h**) 3.8 nm at 1023 K; and (**i**) 3.8 nm at 1123 K under the stress levels of 1.0, 1.4, and 1.8 GPa.

For nanocrystalline materials, vacancy formation is one of the main drivers of diffusion the creep mechanism, owing to the larger volume fraction of grain boundaries in nanocrystalline materials and the higher probability of vacancies at grain boundaries. In order to further explore this mechanism, the vacancy number curve corresponding to the model in Figure 6 has been obtained, as shown in Figure 7. In this study, the defect crystal has been compared with the corresponding perfect lattice, and the vacancy and non-atomic occupied sites are distinguished by employing the Wigner-Seitz cell method.

Comparing Figure 6 and Figure 7, it is noted that although the vacancy number and MSD curves exhibit a similar trend, the time nodes of the curve turning point are different. The observed difference might be explained as follows: as the creep deformation progresses in TiAl alloy, the volume fraction of the amorphous structure increases gradually, thus, resulting in a large number of vacancies. In general, the atoms diffuse through the vacancies relatively easily as compared with the diffusion associated with the replacement of other atoms. However, at sufficiently high temperatures and stress levels, the atoms have sufficient energy to break through the barrier and result in lattice diffusion by replacing other atoms. Therefore, the MSD curve not only contains the information about the atom diffusion through vacancy, but also provides insights into the atom diffusion through substitution.

## 4. Conclusions

In this study, the molecular dynamics simulation has been used to analyze the factors affecting the creep properties of TiAl alloy at high temperatures. For this, the different creep stages of TiAl alloy have been studied at a high temperature, and the deformation mechanisms for different creep stages have been explained. The main conclusions are as follows:

(1) The creep properties of TiAl alloy are significantly affected by temperature and stress. The higher the temperature and stress, the higher the steady-state creep rate of TiAl alloy, and the earlier the rapid creep stage is reached. Decreasing grain size also accelerated the creep process.

(2) Evolution and analysis of the crystal defects revealed that the structure of the grain boundary is damaged solely during the primary and steady-state creep stage, and the crystal structure is retained inside the grains. After entering the rapid creep stage, the structure inside the grain is also destroyed, with the amorphous process extending from the grain boundary to the grain interior.

(3) During the steady-state creep phase, the dislocation density of TiAl alloy slowly rises, and the atomic diffusion of the grain boundary can also be observed. It suggests that this phase is associated with dislocation motion and grain boundary diffusion. At the same temperature, the higher the stress, the larger its steady-state creep rate and the higher the dislocation density. It shows that the steady-state creep stage is dominated by the dislocation motion mechanism at high stress, while the diffusion creep mechanism dominates it at low stress. In the rapid creep stage, dislocation is not the dominant factor in the amorphous process of TiAl alloy. In case the diffusion of the atoms along the grain boundary as well as the diffusion within the grain can be observed, it can be suggested that the creep mechanism for this stage is comprised of grain boundary diffusion and lattice diffusion.
